# Surface energies of elemental crystals

**DOI:** 10.1038/sdata.2016.80

**Published:** 2016-09-13

**Authors:** Richard Tran, Zihan Xu, Balachandran Radhakrishnan, Donald Winston, Wenhao Sun, Kristin A. Persson, Shyue Ping Ong

**Affiliations:** 1 Department of NanoEngineering, University of California San Diego, 9500 Gilman Dr, Mail Code 0448, La Jolla, California 92093-0448, USA; 2 Energy Technologies Area, Lawrence Berkeley National Laboratory, Berkeley, California 94720, USA; 3 Department of Materials Science and Engineering, Massachusetts Institute of Technology, Cambridge, Massachusetts 02139, USA; 4 Department of Materials Science & Engineering, University of California, Berkeley, California 94720-1760, USA

**Keywords:** Computational methods, Surfaces, interfaces and thin films, Density functional theory

## Abstract

The surface energy is a fundamental property of the different facets of a crystal that is crucial to the understanding of various phenomena like surface segregation, roughening, catalytic activity, and the crystal’s equilibrium shape. Such surface phenomena are especially important at the nanoscale, where the large surface area to volume ratios lead to properties that are significantly different from the bulk. In this work, we present the largest database of calculated surface energies for elemental crystals to date. This database contains the surface energies of more than 100 polymorphs of about 70 elements, up to a maximum Miller index of two and three for non-cubic and cubic crystals, respectively. Well-known reconstruction schemes are also accounted for. The database is systematically improvable and has been rigorously validated against previous experimental and computational data where available. We will describe the methodology used in constructing the database, and how it can be accessed for further studies and design of materials.

## Background & Summary

The surface properties of a crystal are crucial to the understanding and design of materials for many applications. For instance, technologies such as fuel cells and industrial chemical manufacturing require the use of catalysts to accelerate chemical reactions, which is fundamentally a surface-driven process^1–9^. Surface effects are especially important in nanomaterials, where relatively large surface area to volume ratios lead to properties that differ significantly from the bulk material^10–14^. For example, the nanoscale stability of metastable polymorphs is determined from the competition between surface and bulk energy of the nanoparticle^15–18^.

The stability of a surface is described by its surface energy *γ*, a measure of the excess energy of surface atoms due to a variety of factors, such as the broken bonds yielding undercoordinated atoms. This fundamental quantity is important in understanding surface structure, reconstruction, roughening and the crystal’s equilibrium shape^19^. Despite its importance, experimental determination of surface energies, especially for specific facets, is difficult and rare^20–24^. Furthermore, experimentally observed Wulff shapes are often inconsistent across studies due to the sensitivity of high energy facets to temperature and impurities^25^. References 26 and 20 have accumulated a large set of metallic elemental surface energy data by extrapolating surface tension of liquid phases for solid surfaces. Reviews of such surface tension techniques have been compiled by Mills *et al.*^27^ and Keene^28^.

First principles computations such as those based on density functional theory (DFT) methods are important complementary tools to experimental techniques in characterizing surface properties of a material^29–31^. Computational techniques provide the means to precisely control the surface structure and composition. Indeed, fundamental and application-driven computational studies of surfaces in the literature are extensive^8,32–34^. On the broader scale, Vitos *et al.*^35^ have previously compiled a database of surface energies for all metals up to Pu using the full charge density (FCD) DFT method, a technique based on the coupling of the linear muffin-tin orbital method and the atomic-sphere approximation^36^. However, this database is limited to surfaces of *ground state crystals up to a maximum Miller index (MMI) of 1 only*.

The challenges for DFT determination of surface properties are three-fold. First, the choice of the exchange correlation functional as well as other parameters such as pseudopotentials, integration grid, etc. can significantly affect the accuracy and convergence of surface energies, which in turn leads to values that are generally difficult to compare across different works^37^. Second, the typical ‘slab’ approach for performing surface energy calculations requires the use of large supercells with the introduction of a large vacuum region, which makes such calculations computationally intensive, especially for low symmetry materials and high Miller indices. Finally, some surfaces undergo significant reconstruction to reduce surface energy and increase stability, the most notorious example being the 7×7 reconstruction of the Si (111) surface^38–40^. In order for the DFT geometry optimization algorithms to identify the global energy minimum surface structure, a reasonable initial guess for the reconstructed surface is needed to avoid relaxation to a local minimum, and such initial guesses are difficult to determine *a priori* without experimental input.

In this work, we present a large database of surface energies and Wulff shapes of more than 100 polymorphs of about 70 elements constructed using high-throughput DFT calculations. The elemental crystals studied include all polymorphs available in the Materials Project (https://www.materialsproject.org)^41^ and the chemistries covered include both metals and non-metals. All surfaces up to a maximum absolute Miller index (MMI) of 2 and 3 were computed for non-cubic and cubic crystals respectively. To address the first and second challenges outlined above, a robust computational workflow based on the efficient convergence scheme proposed by Sun *et al.*^42^ was developed to automate the large number of multi-step calculations. To partially address the third challenge, we include well-known reconstructions such as the missing row (110) and hexagonal (111) reconstructions for the face-centered-cubic surfaces^43,44^ in our data set. All data have been made publicly available via the Crystalium web application at the Materials Virtual Lab website (http://crystalium.materialsvirtuallab.org), as well as the Materials Project’s graphical user interface and RESTful Application Programming Interface (API)^41,45^. The full dataset is also available as a JSON file at the Dryad-repository (Data Citation 1). We show that the calculated surface energies are in excellent agreement with available experimental values. We will also provide an assessment of the effect of surface reconstructions on the accuracy of the data.

## Methods

### Slab model generation

**Figure 1:**
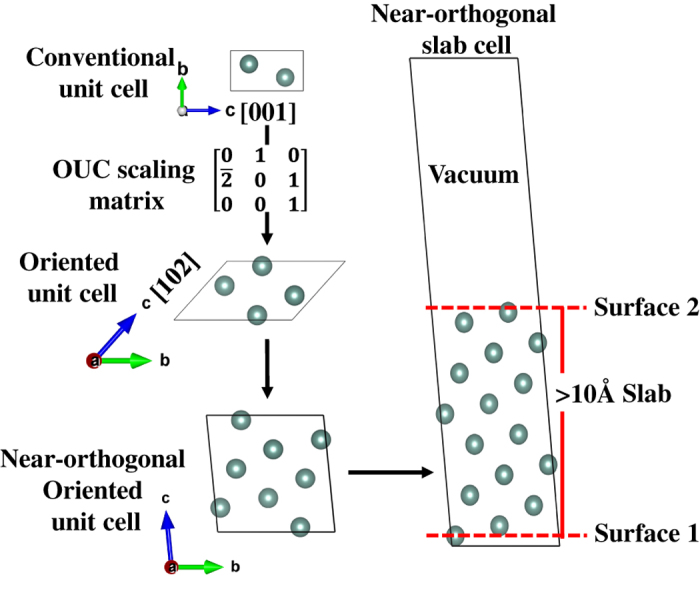
Slab construction. Construction of Y $(10\bar{1}2)$ slab from the conventional unit cell. Note that the c lattice vector does not necessarily need to be perpendicular.

The approach used to compute surface energies in this work is the typical slab model, wherein a supercell of the crystal oriented to expose the relevant surface of interest is generated, and atoms are removed from a portion of the supercell to create a vacuum. An example is given in Fig. 1. Starting from the conventional unit cell, a series of lattice vector transformations is performed to create an ‘oriented’ unit cell (OUC) where the **a** and **b** lattice vectors are parallel to the plane with Miller indices (*hkl*) and the **c** lattice vector is not parallel to the plane. It should be noted that *c* vector is not necessarily perpendicular to the plane^42^, though the ‘most orthogonal’ vector that can be obtained within a reasonable cell size is used. The OUC is then extended by multiples of the **c** lattice vector and atoms are removed to generate the slab model with a desired slab and vacuum thickness. To obtain all symmetrically distinct terminations for a given plane, atoms in the OUC are shifted in the **c** direction prior to extension to generate the slab model. All slabs generated are constrained to have symmetric top and bottom surfaces. The model generation algorithm has been implemented in the Python Materials Genomics (pymatgen) materials analysis library^46^, with a comprehensive set of unit tests to ensure robust functioning of the code.

### Computational methodology

For a given slab model of a facet with Miller index (*hkl*), the surface energy $\gamma_{hkl}^{\sigma }$ is given by the following expression:

(1)γhklσ=Eslabhkl,σ−Ebulkhkl×nslab2×Aslab

where, $E_{slab}^{hkl,\sigma }$ is the total energy of the slab model with termination *σ*, $E_{bulk}^{hkl}$ is the energy per atom of the bulk OUC, *n*_*slab*_ is the total number of atoms in the slab structure, *A*_*slab*_ is the surface area of the slab structure, and the factor of 2 in the denominator accounts for the two surfaces in the slab model. To efficiently converge the surface energy, both the bulk and slab energies were calculated using the OUC, which allows consistent reciprocal integration grids to be used for the bulk and slab calculations^42,47^. The OUC was fully relaxed in both cell volume and atomic positions, while the slabs were relaxed in the atomic positions only.

All DFT^48,49^ energy calculations were performed using the Vienna Ab initio Simulation Package (VASP)^50–52^ within the projector augmented wave (PAW)^53^ approach. The exchange-correlation effects were modeled using the Perdew-Berke-Ernzerhof (PBE) generalized gradient approximation (GGA)^54^ functional, and all calculations were spin-polarized with a plane wave cutoff energy of 400 eV. The pseudopotentials used are similar to those used in the Materials Project^46^. The energies and atomic forces of all calculations were converged to within 10^−6^ eV and 0.01 eV Å^−1^ respectively. The Methfessel-Paxton method^55^ was chosen as the smearing algorithm, the blocked Davidson iteration scheme^56^ was chosen as the electron minimization algorithm, and ions were updated using the conjugated gradient algorithm. Γ-centered *k*-point meshes of $\frac{50}{a}\times \frac{50}{b}\times \frac{50}{c}$ and $\frac{50}{a}\times \frac{50}{b}\times 1$ were used for OUC and slab calculations respectively with non-integer values rounded up to the nearest integer. Through a series of comprehensive convergence tests, it was determined that vacuum and slab thicknesses of at least 10 Å were sufficient to ensure that the surface energies were converged to within 0.02 J*m*^−2^.

### Wulff shape

**Figure 2:**
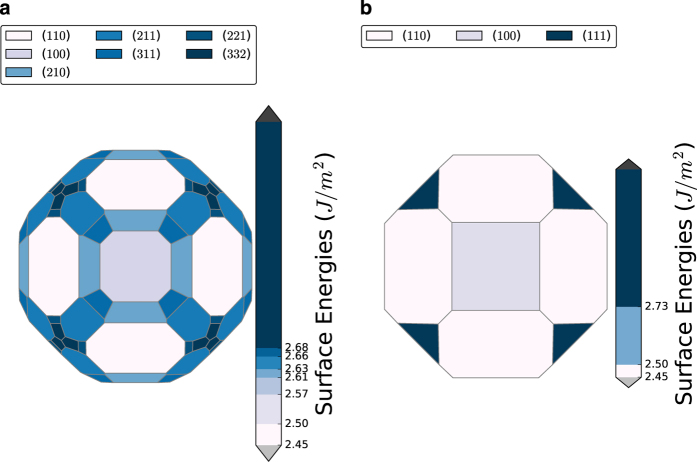
Wulff shape of Fe. The Wulff shape of *α*-Fe generated with surface energies for facets up to a max Miller index of (**a**) 3 and (**b**) 1.

The Wulff construction gives the crystal shape under equilibrium conditions^19,57,58^. In this construction, the distance of a facet from the crystal center is proportional to the surface energy of that facet, and the inner convex hull of all facets form the Wulff shape. Here, we use the methodology developed by Miracle-Sole^58^ to construct the Wulff shape. An example of the Wulff shape of Fe is given in Fig. 2a, which contains all surfaces up to a MMI of 3. To illustrate the importance of sampling higher Miller index surfaces in obtaining an accurate Wulff shape, the Wulff shape of Fe up to MMI of 1 is shown in Fig. 2b. It is evident that inclusion of higher-index surfaces is necessary to produce an accurate description of the equilibrium crystal shape.

The area fraction $f_{hkl}^{A}$ of each symmetrically distinct facet under equilibrium conditions can be determined from the Wulff shape. We define the weighted surface energy $\bar{\gamma }$ using this fraction as given by the following equation:

(2)γ¯=∑{hkl}γhklAhklΣAhkl=∑{hkl}γhklfhklA

where *γ*_*hkl*_ is the surface energy of a unique facet existing in the Wulff shape, *A*_*hkl*_ is the total area of all facets in the {*hkl*} family in the Wulff shape, and $f_{hkl}^{A}$ is the area fraction of the {*hkl*} family in the Wulff shape. In this work, $\bar{\gamma }$ is used as a basis for comparison to experimentally determined surface energies, for which usually only one value is reported.

### Anisotropy measures

Several measures of surface anisotropy have been proposed in the literature. For instance, the ratio *γ*_111_/*γ*_110_ is frequently used for fcc and bcc metals, but such surface-specific measures lack general applicability across crystals of different symmetry. The most commonly used general measure of anisotropy is the shape factor *η*, which is given by the following equation:

(3)η=AV2/3

where *A* and *V* are the surface area and volume of the Wulff shape, respectively. The shape factor is a useful quantity in determining the critical nucleus size^19^. Typically, the shape factor is compared against that of an ideal sphere $\left(\eta ={(36\pi )}^{\frac{1}{3}}\right)$, and a larger *η* indicates greater anisotropy.

Though generally applicable, *η* does not account for variation in surface energies except indirectly through its impact on the Wulff shape. In the database, we have provided an alternative definition of surface energy anisotropy *α*_*γ*_ given by the following equation:

(4)αγ=∑{hkl}(γhkl−γ¯)2fhklAγ¯

*α*_*γ*_ can effectively be viewed as a coefficient of variation of surface energies that is normalized for comparison across crystals with different weighted surface energies. A perfectly isotropic crystal would have *α*_*γ*_=0. *α*_*γ*_ is comparable across different crystal systems and accounts for all surfaces based on their relative importance (in terms of contribution to the Wulff shape).

### Data scope

The current release of the database contains surface energies and Wulff shapes of more than 100 polymorphs of about 70 elements. This database is far larger in scope than previous compilations of surface energies in several important ways:

1. Both metals and non-metals are included.
2. All polymorphs for each element available in the Materials Project database are included. This is in contrast with previous works where only the most stable ground state crystal structures were studied.
3. All surfaces up to a maximum Miller index (MMI) of two and three were calculated for all non-cubic and cubic crystals respectively. The most common spacegroups among the crystals sampled are $Fm\bar{3}m$, $Im\bar{3}m$, *P*6_3_/*mmc*, and $Fd\bar{3}m$, which contains 13, 13, 12, and 13 symmetrically distinct surfaces respectively up to the MMI used in this work. The only exceptions are the ground state crystals of Mn, S, P, Se and B for which only the terminations and surfaces containing the least number of broken bonds were calculated due to the low symmetry and/or large unit cell sizes.
4. Well-known reconstruction schemes are incorporated. At the time of writing, the schemes considered are the $Fm\bar{3}m$ (110) 2×1 missing row and (111) 1×1 hexagonal and $Fd\bar{3}m$ (111) 2×1, (110) 1×1 and (100)p 2×1 reconstructions.

This database will be systematically improved through continuous updates. Future updates will include higher MMIs and other surface reconstruction schemes such as the well-known fcc(100)-hex surfaces^59^. Further expansion to non-elements, e.g., binaries, ternaries, etc. is also planned at a later stage.

### Surface calculation workflow

**Figure 3:**
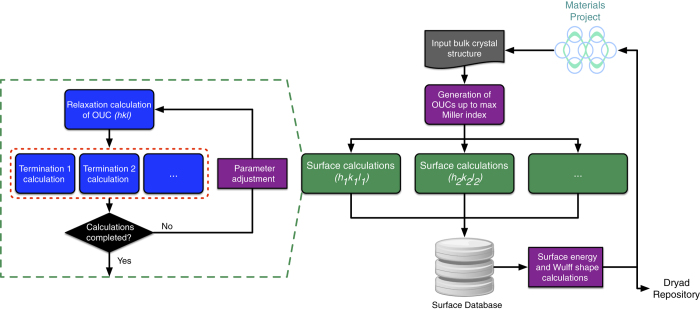
High throughput workflow. A schematic of the high throughput-infrastructure for the calculation the surface energies of elemental crystalline solids. Dashed blocks represent workflow steps performed in parallel.

Figure 3 shows an outline of the high-throughput workflow used in this work implemented using the FireWorks software package^60^. The initial bulk crystals were obtained by querying for all elemental crystals from the Materials Project via the Materials API^45^. All OUCs up to the MMI for each crystal were then automatically generated. For each OUC (corresponding to a specific Miller index (*hkl*)), a full relaxation was then carried out, and the slab models for distinct terminations were then generated from the fully relaxed OUC and calculated. To handle errors that may arise during calculations, the custodian software package^46^ was used as a wrapper around VASP together with a set of robust error handling rules. The results from successful calculations were then automatically inserted into a MongoDB document-based database. The metadata of the DFT calculations and the surface properties extracted (see Tables 1 and 2) were subsequently inserted into the Materials Project database and the Dryad repository.

Consistency checks were devised as part of the workflow to detect possible errors and anomalous behavior in the surface calculations. Calculations containing such anomalies are tagged with a warning containing a list of their brief descriptions. The nature of these warnings are detailed in Table 3. Entries tagged with such warnings do not automatically indicate that the calculations are invalid. For example, although surface atom displacements on relaxation are typically expected to be less than 5% (ref. 33), the relaxation of the (110) surface of diamond Si exceeds this value due to its tendency to reconstruct to achieve stability.

### Code availability

Pymatgen is the primary materials analysis code used in this work, and the surface construction and input file generation algorithms are implemented in this package. Both pymatgen and the custodian error recovery library are open-source software under the MIT (Massachusetts Institute of Technology) License. The high-throughput workflow was implemented using the FireWorks library, which is freely accessible under a modified GPL (GNU General Public License)^60^. All implemented algorithms, including the model generation and Wulff shape calculation, come with a comprehensive set of unit tests. The open-source software codes are also continuously tested via a continuous integration service. The VASP DFT code used is copyrighted by the University of Vienna, Austria and is accessible under a paid license.

## Data Records

**Figure 4:**
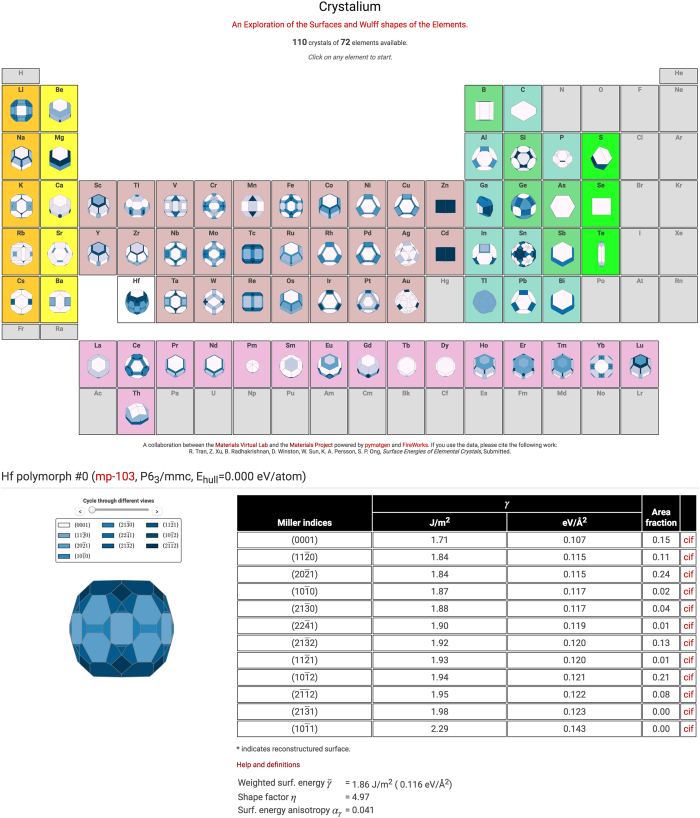
Crystalium web application. Screenshot of the Crystalium web application.

A user-friendly web application, Crystalium, has been developed to allow users to efficiently explore the Wulff shapes, surface energies and slab structures of the polymorphs investigated. This web application is at http://crystalium.materialsvirtuallab.org (a screenshot is given in Fig. 4). In addition, the surface energies and Wulff shapes are also available on the Materials Project website (https://www.materialsproject.org) on the detailed data pages of specific crystals. Two JSON data files are also available in a Dryad repository (Data Citation 1). The first data file contains the complete set of metadata and surface properties for all materials studied so far while the second contains the key VASP input parameters and output data for all slab calculations performed in this study.

### File format

The surface properties for each material is stored as an individual JSON document (Data Citation 1). The material is described by its metadata which contains information such as the spacegroup, formula, a unique Materials Project identifier (mp-id), the energy per atom above the hull (a measure of the relative stability of the bulk crystal) and the polymorph number. A description of the metadata can be found in Table 1. A similar JSON document provides the key VASP input parameters and output data for each slab calculation, which is also uniquely identified using the mp-id of the crystal.

### Properties

The JSON document for each entry contains an organized list of sub-entries that describes the properties of each surface in detail. Each sub-entry contains information such as the Miller index, surface energy and the fraction of the Wulff shape’s surface area occupied by this facet. For each Miller index, the lowest surface energy termination, including among different reconstructions investigated where applicable, is provided in each sub-entry. The slab structure used to model the surface is available as a string in the JSON document in the format of a Crystallographic Information File (cif), which can also be downloaded via the Materials Project website and Crystalium web application. In addition, the weighted surface energy (equation (2)), shape factor (equation (3)), and surface anisotropy (equation (4)) are given. Table 2 provides a full description of all properties available in each entry as well as their corresponding JSON key.

## Technical Validation

The data was validated through an extensive comparison with surface energies from experiments and other DFT studies in the literature. Due to limitations in the available literature, only the data on ground state phases were compared.

### Comparison to experimental measurements

**Figure 5:**
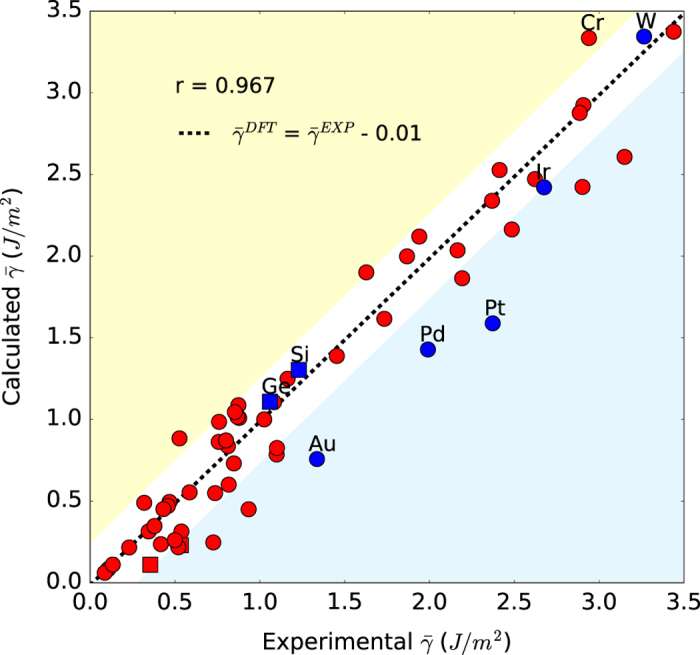
Comparison to experimental surface energies. Plot of experimental versus calculated weighted surface energies for ground-state elemental crystals. Structures known to reconstruct have blue data points while square data points correspond to non-metals. Points that are within the standard error of the estimate (±0.27 J m^−2^) lie in the white region.

Experimental determination of surface energy typically involves measuring the liquid surface tension and solid-liquid interfacial energy of the material^20^ to estimate the solid surface energy at the melting temperature, which is then extrapolated to 0 K under isotropic approximations. Surface energies for individual crystal facets are rarely available experimentally. Figure 5 compares the weighted surface energies of all crystals (equation (2)) to experimental values in the literature^20,23,26–28^. It should be noted that we have adopted the latest experimental values available for comparison, i.e., values were obtained from the 2016 review by Mills *et al.*^27^, followed by Keene^28^, and finally Niessen *et al.*^26^ and Miller and Tyson^20^. A one-factor linear regression line ${\bar{\gamma }}^{DFT}={\bar{\gamma }}^{EXP}+c$ was fitted for the data points. The choice of the one factor fit is motivated by the fact that standard broken bond models show that there is a direct relationship between surface energies and cohesive energies, and previous studies have found no evidence that DFT errors in the cohesive energy scale with the magnitude of the cohesive energy itself^61^.

We find that the DFT weighted surface energies are in excellent agreement with experimental values, with an average underestimation of only 0.01 J m^−2^ and a standard error of the estimate (SEE) of 0.27 J m^−2^. The Pearson correlation coefficient *r* is 0.966. Crystals with surfaces that are well-known to undergo significant reconstruction tend to have errors in weighted surface energies that are larger than the SEE.

The differences between the calculated and experimental surface energies can be attributed to three main factors. First, there are uncertainties in the experimental surface energies. The experimental values derived by Miller and Tyson^20^ are extrapolations from extreme temperatures beyond the melting point. The surface energy of Ge, Si^62^, Te^63^, and Se^64^ were determined at 77, 77, 432 and 313 K respectively while the energy of the (0001) surface of pyrolytic graphite was determined using the sessile drop technique under high temperatures^65^. In addition, the possibility of contamination by surface active elements such as oxygen can lead to lower surface tension values. As a result, the higher value of surface tension measured in experiments is often argued to be the more accurate value^27,28^.

Second, the limitations of the exchange-correlation functional used can also cause discrepancies^35,66^. Though the average difference between the computed PBE surface energies in this work and experimental surface energies is very small (0.01 J m^−2^), there is nevertheless non-negligible differences for specific elements. In addition, this study does not take into consideration the effects of Van der Waals forces in materials such as graphite where it is the dominant interaction between graphene layers in the (0001) direction. Of the 1,000 different surfaces studied, only the (0001) surfaces of the two graphite polymorphs have unphysical negative surface energies, which was also previously observed by Ooi *et al.*^67^.

**Figure 6:**
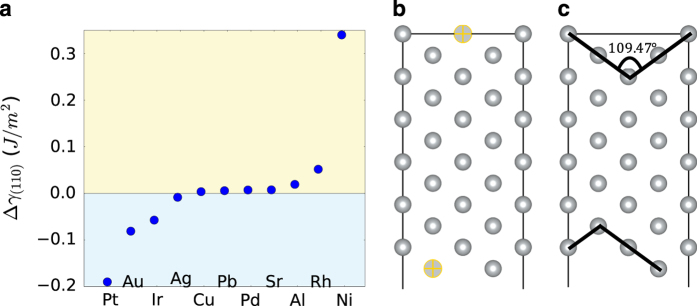
Fcc (110) surface reconstruction. Comparison of the reconstructed and unreconstructed (110) surfaces for fcc materials. (**a**) Plot of the difference in surface energy $\left(\Delta \gamma_{110}=\gamma_{110}^{\mathrm{reconstruct}}-\gamma_{110}^{\mathrm{relaxed}}\right)$ between the reconstructed and unreconstructed (110) surface of fcc metals. Negative values indicate a tendency to reconstruct. (**b**) Unreconstructed and (**c**) reconstructed models for a (110) fcc slab are shown.

Finally, surface reconstructions could also contribute to differences between the computed and experimental values. To understand the effect of reconstructions on surface energies, we compared the surface energy of the relaxed (110) fcc metal surfaces from the database to the reconstructed configuration described by Zhang *et al.*^43^, as shown in Fig. 6. We find that the missing row reconstruction is predicted to be favored (lower in energy) over the unreconstructed surface for Pt, Au and Ir only, in agreement with previous experimental and computational results^43,68^. Even for the surfaces that undergo reconstruction, the differences between the reconstructed and unreconstructed surface energies are relatively small (<0.2 J m^−2^) in metals.

Exceptional cases of reconstruction with large differences between the relaxed and reconstructed surface energies do exist. Semiconductors such as Si and Ge are known to have significantly lower surface energies in their reconstructed state. The experimental, reconstructed and relaxed surface energies for Si are shown in Table 4. The reconstructed surface energies are much closer to those found experimentally than the unreconstructed surface energies. The reconstructed (111) surface in particular shows the largest energy difference (0.85 J m^−2^). It should be noted that this HT work did not explore the well-known 7×7 reconstruction^69,70^ of the Si(111) surface due to the large supercell required.

### Comparison with previous computational studies

Table 5 presents a comparison of the calculated surface energies in this work with those computed in previous works. Unlike the comparison with experimental data, surface-specific comparisons are presented to demonstrate the accuracy of the data. An ‘averaged’ experimental value is provided where available for reference. Despite the fact that the literature values come from studies with widely different parameters, including choice of exchange-correlation functional, pseudopotential choice/full electron calculations, etc., we find that the calculated values in this work differ from these literature values by only 2–13% (refs 25,71). The (111) surface of fcc Pt from Da Silva *et al.*^71^ has the largest (13%) difference. This is largely due to use of pseudopotentials in this study while Da Silva *et al.*^71^ used an all-electron method.

In general, we find that the calculated surface energies in this work are slightly lower than that in the work of Vitos *et al.*^35^ This observation may be attributed to the atomic sphere approximation used in the FCD calculations by Vitos *et al.*^35^, which is known to hinder relaxation at the surface, thus leading to higher surface energy values^71^. Also, our calculated surface energies are significantly lower than those computed using the local density approximation (LDA) functional in the literature, which may be attributed to the LDA functional’s propensity to overbind compared to the GGA functional used in this work^72–75^.

The planes with the highest atomic density per unit area of fcc, bcc and hcp materials are the {111}, {110} and {0001} planes respectively. According to the classic broken bond model, the minimization of broken bonds in these surfaces leads to these surfaces having the lowest *γ*^76,77^. Our results support this empirical model, with a few notable exceptions. For example, the (0001) surfaces for hcp Sc and Y and the (110) surface for Li are not the lowest energy surfaces among the facets investigated. These exceptions are also observed in Vitos *et al.*^35^ for various materials and in other previous first-principle studies for the Li (110) surface^35,78,79^.

## Usage Notes

The database in this study is the most extensive collection of calculated surface energies for elemental crystalline solids to-date. When used with data mining and machine learning techniques, the database can be used to reveal trends in surface phenomena and guide the screening of potentially interesting materials for target surface properties. For instance, the relaxed surface structures and energies of many metals, particularly the noble metals, will be highly useful in the study of surface absorption of molecules, which is of great fundamental relevance in catalysis. Consideration of surfaces is especially crucial in nanomaterials design, where surface effects tend to dominate the overall performance and properties. We also anticipate the data presented to be a useful starting point for the study of the interfaces between materials, either within the same material (e.g., grain boundaries) or between different materials. In the near future, we will provide the facility for Materials Project users to upload experimentally proposed reconstructions to further improve the completeness of the database. Furthermore, future enhancements would include the surface properties of not just elements, but also multicomponent compounds (including alloys).

## Additional Information

**How to cite this article:** Tran, R. *et al.* Surface energies of elemental crystals. *Sci. Data* 3:160080 doi: 10.1038/sdata.2016.80 (2016).

## Supplementary Material



## Figures and Tables

**Table 1:** The meta data for a particular material.

**Key**	**Datatype**	**Description**
formula	string	Chemical formula
material_id	string	IDs for entries in Materials Project
polymorph	integer	Rank of polymorph stability (0 being ground state)
spacegroup	string	International spacegroup symbol and number
e_above_hull	float	Energy above the hull reported by Materials Project.

**Table 2:** Surface properties for a crystal.

**Property**	**Key**	**Datatype**	**Unit**	**Description**
Weighted surface energy, $\bar{\gamma }$	weighted_surface_energy	float	J m^−2^	Surface energy weighted by the Wulff shape’s facet areas.
	weighted_surface_energy_EV_PER_ANG2		eV Å^−2^	
Anisotropy, *α*_*γ*_	surface_anisotropy	float		Measure of the anisotropy of the surface energies weighted by relative importance.
Shape factor, *η*	shape_factor	float		Measure of the anisotropy based on the geometry of the Wulff shape.
Surfaces	surfaces	list		List of sub-entries describing an individual surface.
Surface energy, *γ*_hkl_*	surface_energy	float	J m^−2^	Surface energy corresponding to the most stable termination or reconstruction.
	surface_energy_EV_PER_ANG2		eV Å^−2^	
Miller index (*hkl*)*	miller_index	list		Miller index of the slab.
Task ID*	tasks	int		Task ID of the OUC and slab calculation the sub-entry properties were derived from.
Reconstruction*	is_reconstructed	boolean		Indicates whether the sub-entry properties corresponds to a reconstructed slab
Wulff surface area fraction*	area_fraction	float		Fraction of the Wulff shape’s surface area occupied by surface’s facets.
Slab structure*	structure	string		Slab used to model the surface as a Crystallographic Information File (cif).
Properties denoted by * are defined for each distinct surface.				

**Table 3:** The possible warnings tagged for each surface calculation.

**Warning name**	**Description**
|*bulk*_*vol*_*rel*| > 1%	Relaxation of the OUC volume is greater than 1%.
|*slab*_*site*_*rel*| > 10%	Relaxation of the slab sites is greater than 10%.
|*slab*_*site*_*rel*| > 5%	Relaxation of the slab sites is greater than 5%.
negative_surface_energy	The surface energy is negative.

**Table 4:** Comparison between the calculated unreconstructed and reconstructed surface energies, and experimental surface energies (in J m^−2^) for various surfaces of Si.

**Miller index**	**Unreconstructed**		**Reconstructed**		**Experiment**	
	***γ***	$f_{hkl}^{A}$	***γ***	$f_{hkl}^{A}$	***γ***	**Reconstruction**
(111)	1.57	0.09	1.30	0.45	1.23	(2×1)
(110)	1.76	0.00	1.51	0.00	1.43	(1×1)
(100)	2.13	0.00	1.28	0.36	1.36	*p* (2×1)
The calculated area fractions based on the Wulff shapes are also provided.						

**Table 5:** A comparison of the high-throughput values to experimental and computed values for materials from the literature

**Material**	**Surface**	**Surface energy** *γ* **(J m**^**−2**^)		
		**This work**	**Prev. DFT**	**Experimental**
Ni	(110)	2.29	2.37 (ref. 35)–2.31 (ref. 25)	2.44* (ref. 27)
	(210)	2.4	2.43 (ref. 25)	
	(100)	2.21	2.25 (ref. 25)–2.43 (ref. 35)	
	(221)	2.17	2.2 (ref. 25)	
	(111)	1.92	2.01 (ref. 35)–1.95 (ref. 25)	
Mg	$\left(10\bar{1}0\right)$	0.6	0.78 (ref. 35)	0.82* (ref. 28)
	(0001)	0.54	0.54 (ref. 71)–0.79 (ref. 35)	
Ba	(110)	0.31	0.38 (ref. 35)–0.37 (ref. 80)	0.34* (ref. 28)
	(100)	0.32	0.37 (ref. 80)–0.35 (ref. 35)	
	(111)	0.39	0.45 (ref. 80)–0.4 (ref. 35)	
Pt	(110)	1.68	2.91 (ref. 34)–2.27 (ref. 35)	2.37* (ref. 28)
	(100)	1.84	2.73 (ref. 35)–2.23 (ref. 80)	
	(111)	1.48	2.35* (ref. 71)–1.47 (ref. 71)	
Sr	(110)	0.41	0.47 (ref. 80)–0.43 (ref. 35)	0.38* (ref. 28)
	(100)	0.35	0.41 (ref. 35)–0.39 (ref. 80)	
	(111)	0.34	0.5 (ref. 81)–0.4 (ref. 80)	
Mo	(110)	2.8	2.92 (ref. 82)–3.69 (ref. 81)	2.07* (ref. 27)
	(100)	3.18	3.34 (ref. 82)–3.84 (ref. 35)	
	(211)	3.4	3.11 (ref. 82)–3.6 (ref. 35)	
	(111)	2.96	3.24 (ref. 82)–3.74 (ref. 35)	
Bi	(0001)	0.17	NA	0.43* (ref. 28)
Li	(110)	0.5	0.56* (ref. 35)–0.3 (ref. 78)	0.7* (ref. 28)
	(100)	0.46	0.52 (ref. 35)–0.32 (ref. 78)	
	(111)	0.54	0.62* (ref. 35)–0.34 (ref. 78)	
Pb	(100)	0.28	0.64* (ref. 37)–0.32 (ref. 37)	0.52* (ref. 28)
	(110)	0.33	0.72 (ref. 34)–0.33 (ref. 35)	
	(111)	0.25	0.6 (ref. 34)–0.26* (ref. 37)	
Re	(0001)	2.58	4.21 (ref. 35)	2.52* (ref. 27)
	$\left(10\bar{1}0\right)$	2.86	4.63 (ref. 35)	
Ge	(110)	0.97	1.17 (ref. 40)	0.68* (ref. 27)
	(100)	0.87	1.71 (ref. 40)	
	(111)	1.11	1.3 (ref. 40)	
Lu	(0001)	1.13	1.6 (ref. 35)	1.08* (ref. 28)
	$\left(10\bar{1}0\right)$	1.05	1.42 (ref. 35)	
Fe	(110)	2.45	3.0 (ref. 80)–2.43 (ref. 35)	2.41* (ref. 27)
	(100)	2.5	3.12 (ref. 80)–2.22 (ref. 35)	
	(211)	2.61	2.59 (ref. 35)	
	(111)	2.73	3.28 (ref. 80)–2.73 (ref. 35)	
Ga	(001)	0.57	NA	0.02* (ref. 27)
Dy	$\left(10\bar{1}2\right)$	1.0	NA	0.88* (ref. 28)
Sc	$\left(10\bar{1}0\right)$	1.2	1.53 (ref. 35)	1.16* (ref. 28)
	(0001)	1.27	1.83 (ref. 35)	
A range of values is provided based on the lowest and highest values found in the literature. ^37^GGA (Hamann pseudopotentials); ^35^GGA (FCD); ^34^LDA (FP-KKR); ^25^GGA-PBE;				
^40^LDA (Vanderbilt pseudopotentials);				
^80^GGA-PBEsol;				
^71^GGA-PBE (FP-LAPW);				
^81^GGA-PBEsol;				
^82^Local Density Formulation;				
^78^PWGGA (LCGTO);				

*See reference herein.
